# Toxoplasmic Encephalitis Presenting as a Stroke Mimic in an AIDS Patient: A Case Report

**DOI:** 10.7759/cureus.94589

**Published:** 2025-10-14

**Authors:** Alejandra M Rincon-Ponte, Melissa Santibañez, Robert Woods

**Affiliations:** 1 Barry and Judy Silverman College of Pharmacy, Nova Southeastern University, Davie, USA; 2 Department of Pharmacy, Memorial Regional Hospital, Hollywood, USA

**Keywords:** acute stroke mimic, central nervous system infections (cns), cns toxoplasmosis, hiv aids, hospital pharmacy

## Abstract

Toxoplasmic encephalitis (TE), a severe infection caused by Toxoplasma gondii, poses significant risks for immunocompromised patients, particularly those with HIV or AIDS. TE frequently manifests as focal encephalitis with confusion, headache, motor weakness, and fever, which may closely mimic ischemic stroke. These overlapping symptoms create diagnostic challenges, increasing the risk of misdiagnosis and treatment delays, both of which can lead to serious consequences in AIDS patients. This case report discusses the diagnosis and management of TE in a 45-year-old female with AIDS and multiple comorbidities who presented with acute left-sided weakness and facial droop, triggering a stroke alert. The patient, with a critically low CD4 count of 5 cells/μL and a history of toxoplasmosis and Pneumocystis jirovecii pneumonia, exhibited symptoms of left-sided weakness and facial droop. After prompt imaging and review of her extensive medical history, a clinical pharmacist suspected TE as the underlying cause rather than an ischemic stroke. This assessment was supported by her recent visit to an infectious disease specialist, who had treated her for TE the previous month.

This case highlights the critical role of comprehensive evaluations and accurate differential diagnoses by the medical team. Effective TE management depends on early recognition, timely diagnosis, and appropriate therapy initiation. This report underscores the importance of interdisciplinary care, particularly the role of clinical pharmacists in guiding treatment, addressing medication resistance, and promoting adherence to prevent opportunistic infections. In patients with psychosocial barriers to adherence, holistic approaches incorporating psychosocial support and close monitoring are essential for sustained care engagement. Prompt recognition and management of TE can reduce complications and prevent inappropriate interventions, such as unnecessary thrombolytic therapy (e.g., alteplase). By emphasizing early detection, thorough evaluation, and proactive care, healthcare providers can improve outcomes for TE in AIDS patients. This case illustrates the need for vigilance in identifying neurological symptoms and the value of coordinated care to address unmet medical and psychosocial needs in this vulnerable population.

## Introduction

Toxoplasmosis is an infection caused by the intracellular protozoan parasite Toxoplasma gondii (T. gondii) and is associated with clinically significant infection in immunocompromised individuals [1]. Among patients with HIV and/or AIDS, the most common clinical presentation of T. gondii infection is focal encephalitis with headache, confusion, or motor weakness and fever [2]. Primary infection typically occurs through ingestion of tissue cysts in undercooked meat or oocysts (vesicles) shed in cat feces that have sporulated (converted into spores) in the environment [2].

Toxoplasmic encephalitis (TE) is a severe and potentially life-threatening manifestation that presents as an opportunistic infection in AIDS patients with low CD4 counts. The prevalence of TE infection in the HIV/AIDS population in the United States is estimated to be as high as 11% [3] due to non-adherence to antiretroviral therapy (ART), late diagnosis, and viral resistance. However, opportunistic infections, in general, are unlikely in patients maintained on a stable ART regimen, even with CD4 counts <200 cells/μL [4,5], especially if the patient is taking a prophylactic regimen against toxoplasmosis. 

We present a case report describing the clinical manifestation and significance of TE in AIDS, especially its similarity in presentation to an ischemic stroke (e.g., aphasia, one-sided weakness, altered mental status). This presentation can be misdiagnosed as an acute ischemic stroke and may lead to the initiation of inappropriate thrombolysis. This report aims to emphasize the importance of early recognition and management of TE, patient education, adherence to ART and prophylactic antibiotics, and the role of the hospital pharmacist in improving patient outcomes and reducing associated morbidity and mortality. This report was prepared in accordance with the Case Report Statement and Checklist (CARE) guidelines [6].

## Case presentation

Patient information

A 45-year-old African American female (height: 150 cm, weight: 82.7 kg, BMI: 36.76 kg/m^2^) with allergies to dust presented to the emergency department (ED) of a community hospital via emergency medical services after meeting the criteria for a stroke alert. The patient had a medical history significant for HIV/AIDS with a nadir CD4 count of 5 cells/μL, diabetes mellitus type 2, asthma, hyperlipidemia, anxiety, depression, iron deficiency anemia, cervical intraepithelial neoplasia, chronic adenoid hypertrophy, and seizure disorder. She had also experienced an ischemic stroke six years prior, with no residual deficits, and had both a prior toxoplasmosis infection within the same year and a Pneumocystis jirovecii pneumonia (PJP) infection the year prior. Though she had been diagnosed 16 years ago, the hospital’s electronic health records only indicated labs and progress notes from up to 10 years prior. The patient denied smoking or illicit drug use but reported occasional alcohol consumption (a glass of wine). It was not noted if the patient had any pets, including cats, or the nature of her diet and exercise habits. 

The patient had been diagnosed with TE earlier this same year at an office visit with her infectious diseases specialist. She had been prescribed a treatment dose of double-strength sulfamethoxazole/trimethoprim (SMX/TMP) 10 mg/kg twice daily, equivalent to 800/160 mg orally twice daily. A CD4 count had not been taken at that time. She had also been referred to a neurologist, but the patient had failed to follow up. A brain MRI performed one month later had shown no signs of toxoplasmosis. A week before this index ED visit, the patient had visited her infectious diseases specialist with a complaint of numbness and tingling in the left hand, and she had been advised to visit the ED for evaluation of a stroke versus a transient ischemic attack; however, the patient had never presented to the ED for this evaluation.

Clinical findings

Upon arrival to the ED on the index encounter for this report, the patient had a baseline National Institutes of Health Stroke Scale (NIHSS) score of 4 for acute deficits of left-sided weakness and facial droop with a last known normal time of 40 minutes before arrival. She was alert and oriented to person/place/time/situation, and denied any headache, dizziness, dysphagia, or nausea and vomiting. Vital signs were normal except for hypertension (blood pressure: 151/102 mmHg). The patient’s home medication list prior to arrival is summarized in Table 1. 

**Table 1:** Home medication regimen by therapeutic indication, highlighting prophylactic regimens for TE and PJP (relevant to diagnostic considerations) AIDS: acquired immunodeficiency syndrome; HIV: human immunodeficiency virus; MAC: Mycobacterium avium complex; PJP: Pneumocystis jirovecii pneumonia; TE: toxoplasmic encephalitis

Drug and regimen	Indication
Darunavir 800 mg/cobicistat 150 mg/emtricitabine 200 mg/tenofovir alafenamide 10 mg (Symtuza®) 1 tablet by mouth once daily	Antiretroviral therapy (HIV/AIDS)
Sulfamethoxazole/trimethoprim 800-160 mg 1 tablet by mouth twice daily	TE secondary prophylaxis and PJP secondary prophylaxis
Azithromycin 1,200 mg by mouth once weekly	MAC prophylaxis
Aspirin 81 mg by mouth once daily	Secondary stroke prophylaxis
Levetiracetam 500 mg by mouth twice daily	Seizure disorder
Metformin 500 mg by mouth twice daily with meals	Diabetes mellitus type 2
Triamcinolone 0.1% ointment applied topically to lesions on the legs twice daily for 2-4 weeks	Eosinophilic folliculitis (acute condition)
Atorvastatin 20 mg by mouth nightly	Hypertriglyceridemia

Timeline

**Figure 1:**
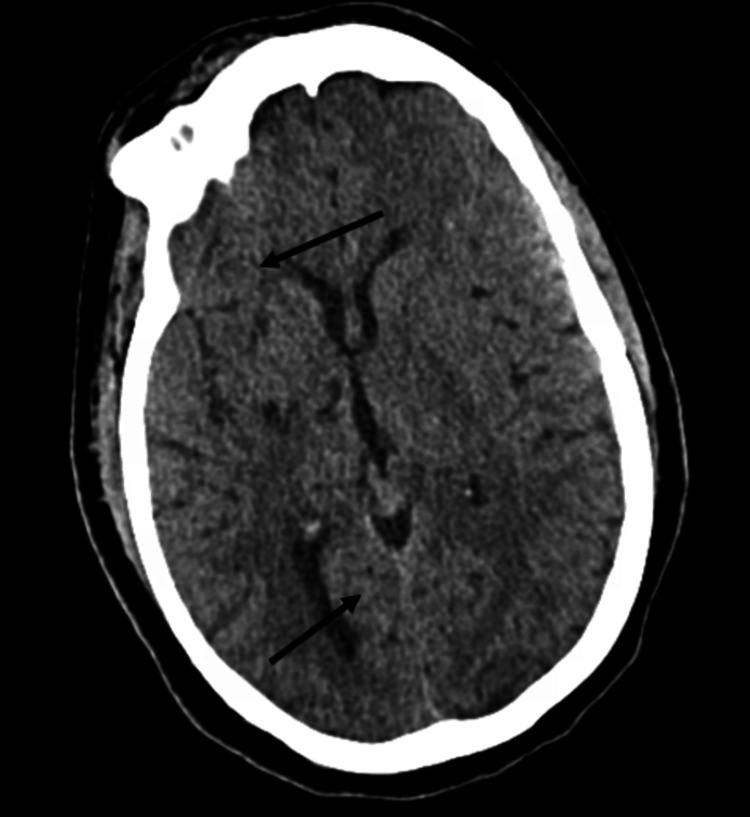
Axial non-contrast CT of the brain The image indicated right frontal vasogenic edema, consistent with opportunistic CNS infection and determined to be evidence of new brain lesions, rather than an acute ischemic infarct. The image also showed development of edema within the right cerebellum with mild mass effect on the fourth ventricle, suggestive of an underlying lesion (lower arrow) in the setting of vasogenic edema in the posterior right frontal lobe in the location of the previously known enhancing lesion (upper arrow), consistent with recurrent toxoplasmosis CT: computed tomography; CNS: central nervous system

The patient was immediately taken for a head CT upon arrival at the ED. Chart review was completed by the clinical pharmacist, who identified that she was being treated prophylactically for toxoplasmosis as well as Mycobacterium avium complex infection with SMX/TMP DS and azithromycin, which was then brought to the attention of the interprofessional care team. Before this, the clinical pharmacist had notified the main pharmacy to pre-mix alteplase for possible acute ischemic stroke, in accordance with hospital policy. The CT (Figure 1) revealed vasogenic edema in the right frontal lobe, suggestive of a new-onset underlying lesion. CT imaging also showed new vasogenic edema in the right cerebellum, suggestive of an underlying lesion and consistent with recurrent toxoplasmosis.

The neurologist, in agreement with the pharmacist and the rest of the care team, ruled out ischemic stroke, and the patient was no longer a candidate for alteplase. The clinical findings suggested a different neurological diagnosis suggestive of TE. The patient was monitored for neurological status changes and admitted to the medical floor. A subsequent brain MRI with and without contrast was ordered for additional diagnostic work-up. 

Diagnostic assessment

**Figure 2:**
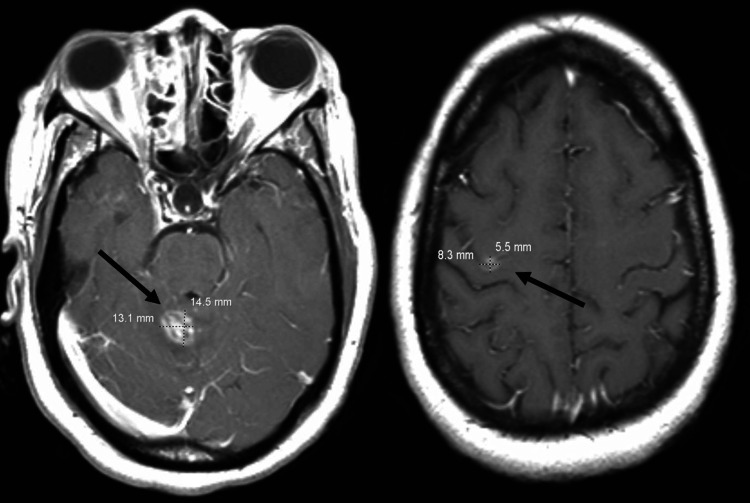
Multiplanar multisequential brain MRI image with and without contrast Imaging indicated a 1.5 x 1.3 x 1.4 cm right superior cerebellum enhancing lesion (left panel) with mild vasogenic edema as well as a 0.6 x 0.8 x 0.6 cm right frontal lobe lesion (right panel) MRI: magnetic resonance imaging

The definitive diagnosis of TE requires a compatible clinical syndrome (typically headache, neurological symptoms, and fever), identification of one or more mass lesions by CT or MRI, and detection of the organism in a clinical sample [3]. The aspects to consider include degree of immunosuppression (e.g., CD4 count), maintenance on a stable ART regimen, and exposure history (e.g., known exposure) [4]. This patient was definitively diagnosed with TE after a brain MRI (Figure 2) revealed new lesions in the right frontal lobe and left cerebellum, demonstrating ring enhancement with associated edema - characteristic of TE and atypical for acute ischemic stroke.

The clinical team’s assessment at this time was multifaceted: left-sided hemiparesis, central nervous system (CNS) toxoplasmosis, and HIV/AIDS non-compliance and acquired resistance to ART. An HIV-1/2 antigen/antibody assay came back reactive, and the HIV-1 antibody test was positive, confirming the HIV diagnosis. Serum anti-Toxoplasma IgG antibodies were positive at >400 IU/mL, and the anti-Toxoplasma IgM antibodies were absent, consistent with a TE diagnosis. A venereal disease research laboratory (VDRL) test was performed alongside a lumbar puncture to obtain cultures and rule out neurosyphilis during this admission. Additional tests included Toxoplasma gondii polymerase chain reaction (PCR) and Epstein-Barr virus (EBV) PCR. The final cultures showed no growth, EBV PCR was 81 IU/mL, and T. gondii DNA was not detected on the PCR two days later. The findings from a lumbar puncture showed a non-traumatic procedure with high CSF protein and leukocytosis with lymphocyte predominance, consistent with acute CNS infection. Table 2 summarizes the patient’s initial diagnostic testing results.

**Table 2:** Diagnostic testing conducted The samples were collected on hospital day 5, and results were obtained on hospital day 12. Positive IgG and negative IgM, consistent with reactivation, rather than new infection HIV: human immunodeficiency virus; IgG: immunoglobulin type G; IgM: immunoglobulin M; PCR: polymerase chain reaction

Diagnostic laboratory test	Result	Reference range
HIV-1/2 antigen/antibody assay	Reactive	Negative
HIV-1 antibody	Positive	Negative
Anti-toxoplasma IgG antibody (blood)	Positive (>400 IU/mL)	Negative
Anti-toxoplasma IgM antibody (blood)	Absent	Negative
Venereal disease research laboratory (lumbar puncture)	No growth	Negative
Epstein-Barr Virus PCR (blood)	81 IU/mL	Negative
T. gondii PCR (blood)	Not detected	Negative
Appearance (lumbar puncture)	Clear/colorless	Clear/colorless
Glucose (lumbar puncture)	51 mg/dL	40-70 mg/dL
Protein (lumbar puncture)	102 mg/dL	15-45 mg/dL
Red blood cells (lumbar puncture)	6 cells/mm^3^	≤10 cells/mm^3^
White blood cells (lumbar puncture)	94 cells/mm^3^	≤5 cells/mm^3^
Lymphocytes (lumbar puncture)	88%	40-80%
Bands (lumbar puncture)	0%	Rare
Segmented neutrophils (lumbar puncture)	7%	0-6%
Eosinophils (lumbar puncture)	0%	Rare
Basophils (lumbar puncture)	0%	Rare
Monocytes (lumbar puncture)	5%	15-45%
Macrophages (lumbar puncture)	Present	≤2%

CSF findings (including the presence of WBC, segmented neutrophils, and monocytes) were consistent with TE and CNS infection due to the newly diagnosed lesions. The patient’s last CD4 count had been done eight months prior and revealed 7 cells/μL. More recently, a nucleic acid test (NAT) had been performed two months ago, revealing a viral load of 30,360 copies/mL. With her significant history of PJP and toxoplasmosis, the patient’s HIV infection had progressed to AIDS. Her HIV genotyping, completed four years prior, showed multiple genetic mutations that had caused her HIV-1 to gain resistance to ART. Her genetic mutations included V90I, I103N, I62V, A71T, K101R, K103N, I178M, S30R, L283I, and E35D. These mutations caused the patient’s virus to be resistant to several antiretroviral drugs listed below: 

K101R and K103N: nevirapine, efavirenz 

I178M: rilpivirine 

Therapeutic intervention

The new frontal lobe lesion identified on MRI was located near the primary motor cortex. The neurologist recommended against a biopsy at the time and instead recommended toxoplasmosis treatment for six weeks and a repeat MRI in two weeks. Darunavir/cobicistat/emtricitabine/tenofovir alafenamide was restarted immediately in the ED and continued while inpatient. For TE treatment, the patient was started on pyrimethamine 200 mg loading dose followed by 75 mg orally daily, sulfadiazine 1500 mg orally every six hours, and leucovorin 25 mg orally daily. Azithromycin prophylaxis was discontinued at this time because MAC was ruled out due to the absence of diagnostic criteria. 

Follow-up and outcomes

During the patient’s eight-day admission, she was seen by neurology, infectious diseases, pulmonary, and neurosurgery services. She tolerated the procedures well and agreed to follow up with her primary care physician (PCP) and infectious diseases specialist. She had a follow-up visit scheduled one month after discharge. The patient missed her post-discharge family medicine appointment despite several phone calls, but she had been regularly following up with the hospital's outpatient specialty pharmacy (for medication refills and adherence check-ins via pillbox reviews) and with infectious diseases. According to the last pharmacy notes from one month post-discharge, medication adherence had improved as evidenced by an empty pillbox with only one missed evening dose of metformin. The patient reported perfect adherence to all components of ART, toxoplasmosis treatments, and other opportunistic infection prophylaxis. She additionally received two new medications (fluconazole 100 mg orally once daily for 10 days for oral thrush and oral dexamethasone 4 mg orally twice daily).

Her medications were being delivered to the infectious diseases physician's office, and she had been compliant. However, approximately two months post-discharge, she required hospital admission for a new seizure episode, for which the working diagnoses included new stroke versus ongoing seizures secondary to the existing brain lesions; these seizures resolved within four days and were ultimately attributed to sequelae from the toxoplasmosis brain lesions. She refused a lumbar puncture, and dexamethasone therapy was stopped. She was discharged on the same ART, with a recommendation to replace the pyrimethamine/sulfadiazine/leucovorin treatment with two SMX/TMP double-strength tablets every 12 hours (5 mg/kg/dose) pending infectious diseases approval, and lacosamide 100 mg orally every 12 hours in addition to levetiracetam for seizure management. 

**Figure 3:**
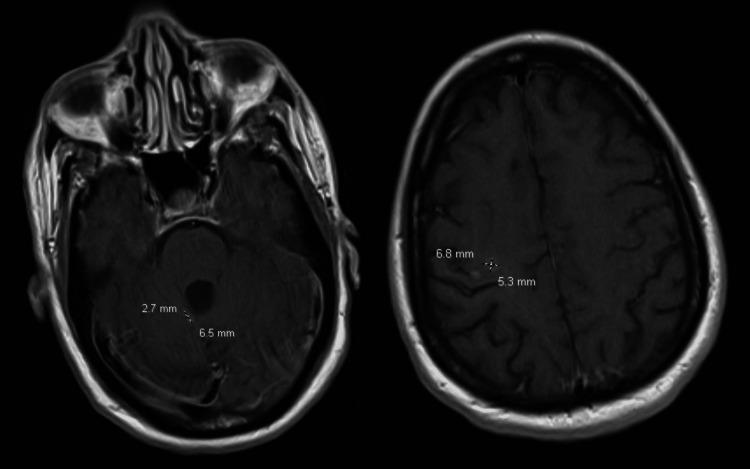
Axial T1 brain MRI images at one year post-admission The image on the left shows the right paravermian cerebellar lesion, and the image on the right shows the posterior right frontal lobe lesion MRI: magnetic resonance imaging

Repeat brain MRI scan obtained one year after this admission showed significant reductions in the existing brain lesions (Figure 3). Specifically, the lesion along the posterior aspect of the right frontal lobe decreased from 14 x 12 mm to 7 x 5 mm, the lesion in the right superior paravermian region of the cerebellum decreased from 16 x 11 mm to 7 x 8 mm, and the 5 mm lesion along the right inferior paravermian region of the cerebellum was no longer visible.

## Discussion

Patients who have completed initial treatment for TE should receive ongoing maintenance therapy, also known as secondary prophylaxis, to prevent recurrence until immune function is restored through ART, at which point discontinuation of treatment may be appropriate [2]. A regimen combining pyrimethamine, sulfadiazine, and leucovorin is highly effective for suppressing TE and also offers protection against PJP [3]. Clinical improvement is typically observed within one to two weeks, during which follow-up imaging should be performed to monitor radiographic changes. [5]. The diagnosis of toxoplasmic encephalitis is confirmed when there is clinical improvement and a reduction in lesion size within two weeks [5]. In this case, the patient’s lumbar puncture results, characterized by elevated CSF protein and lymphocytic predominance, were consistent with an acute CNS infection and aligned with findings commonly reported in TE cases. Combined with the patient’s initial clinical presentation and imaging studies, these findings supported a definitive diagnosis of TE. Follow-up notes from her specialty pharmacy visits confirmed adherence to ART and prophylactic treatment for opportunistic infections. Additionally, a brain MRI performed one year after admission showed decreased lesion sizes, indicating a sustained and favorable response to TE treatment.

Neurological symptoms may serve as cardinal signs of TE. Still, they can also mimic a variety of other neurological conditions, and hence it is important to take the patient’s medical history into account when available. The complexity of toxoplasmosis presentation varies on a case-by-case basis, and in our case, the patient presented with stroke-like symptoms that could have been misinterpreted and caused patient harm. Ultimately, laboratory values from the patient's lumbar puncture samples were consistent with CNS infection and aided in the diagnosis of this TE episode, but given that these final results were not readily available upon initial presentation to the ED, the practical detection of TE as a stroke mimic condition was attributed to the ED pharmacist, whose chart review identified the patient's active TE secondary prophylaxis.

Without these findings from the chart review, alteplase likely would have been administered, as she presented within 3-4.5 hours post-symptom onset and had no contraindications. Because the clinical pharmacist investigated the patient’s prior encounters with infectious disease specialists, and a prompt CT exam was completed, an ischemic stroke was ruled out. Administration of alteplase to patients without an acute ischemic stroke significantly increases their risk of hemorrhagic transformation. Prescribing information for alteplase indicates that symptomatic intracranial hemorrhage occurs in approximately 3.4% of patients [7], underscoring the importance of accurate diagnosis to avoid this life-threatening complication. This adverse effect is critical, with mortality rates in some cases reaching as high as 45% following alteplase-related hemorrhage episode [8].

Because of the mutations previously mentioned, the patient's medication had been changed from Prezcobix® (darunavir/cobicistat) and Tivicay (dolutegravir) to Symtuza® (darunavir/cobicistat/emtricitabine/tenofovir alafenamide) four years before admission. However, due to her most recent CD4 levels, it is evident that the patient had not been compliant with darunavir/cobicistat/emtricitabine/tenofovir alafenamide, enabling her infection to progress further into AIDS, deteriorating her immune system, and potentially causing new mutations. Though the patient’s history of non-adherence and missed appointments may lead to difficulty in establishing a continuum of care, there are gaps in the patient’s care that have not been addressed. The progression of this patient’s disease calls for rechecking of viral load and CD4 count every three to six months. 

Considering the mutated strain of HIV infecting the patient, transitioning from darunavir/cobicistat/emtricitabine/tenofovir alafenamide to a regimen with a different mechanism of action could potentially reduce the risk of resistance and better address the evolving viral profile. Adding the capsid inhibitor, Sunlenca® (lenacapavir), may help decrease the rate of resistance. Lenacapavir is indicated for heavily treatment-experienced adults with multidrug-resistant HIV-1 infection failing their current antiretroviral regimen due to resistance, and, as reported in the CAPELLA trial [9], it is safe and effective for people living with HIV/AIDS who have acquired mutations to ≥2 ARV classes. 

The challenge in our patient’s therapeutic medication management stems from frequently missed critical checkpoints for follow-up testing and visits with her care team, and hence, the opportunity to assess clinical efficacy is missed. Per physicians’ notes, the patient’s non-compliance is presumed to be due to her anxiety and depression symptoms, debilitating her to the point of apathy regarding her ART or prophylactic antibiotics. Her family is actively involved in her life; however, they may inadvertently contribute to her stress and challenges in managing healthcare. There is also a suspicion of impoverishment and transportation issues as social determinants of health that can make it difficult for the patient to refill her ART and antibiotics and miss her doctors’ appointments. To remedy this, the patient was assigned a social worker to help her keep up with her appointments and prescriptions. Outpatient clinic notes document repeated attempts at motivational interviewing to engage the patient in adherence to her care, though this remained a significant barrier due to her underlying anxiety and depression. Adding another behavioral health appointment would likely have been ineffective. However, incorporating proactive, targeted pharmacist interventions, such as adherence counseling and care team coordination, could have significantly improved her overall healthcare management. 

Given the intricate nature of HIV/AIDS management, comprehensive patient and family education is paramount to ensure retention in care. Without sustained engagement, efforts to combat the disease may be futile. This underscores the need for innovative diagnostic methods and personalized treatment plans developed through interdisciplinary collaboration. By fostering a synergistic healthcare environment, we can better navigate the complexities of HIV/AIDS and opportunistic infections like TE, ultimately improving patient outcomes and advancing public health. The integration of cutting-edge research and clinical practice is essential in forging effective, patient-centric care regimens that are both safe and feasible for long-term adherence. To retain patients in care, they must be educated on the importance of adherence to medication therapy and office visits with their care team. Potential barriers to adherence must be identified and targeted specifically to improve compliance, and resources must be provided to the patient and any family members in charge of their care or in their household. A good patient-provider relationship is essential to improving patient outcomes. 

## Conclusions

This report highlights the diagnostic complexities of TE in HIV/AIDS patients, emphasizing key challenges such as adherence, timely recognition of symptoms, and comprehensive disease management. The report also underscores the indistinct clinical presentation of TE and emphasizes the need for rapid prevention and treatment, given the concurrent complexities of HIV/AIDS. To enhance patient outcomes, clinicians must adopt a holistic approach, thoroughly reviewing medical histories for informed decision-making. The neurological manifestations of TE, often mimicking other neurological conditions such as ischemic stroke, require heightened vigilance to avoid misdiagnoses, inappropriate or potentially harmful treatments, and resource misallocation. Medication adherence should be emphasized with all patients. While this single case report underscores the diagnostic challenges and importance of multidisciplinary care, further studies are needed to gain deeper insights into the impact of early recognition and psychosocial support interventions on long-term outcomes in patients with TE and HIV/AIDS.
